# Fibromuscular Dysplasia and Intracranial Aneurysms: A Narrative Review of a Dangerous and Underestimated Association

**DOI:** 10.3390/jcm14228080

**Published:** 2025-11-14

**Authors:** Marialuisa Zedde, Maria Simona Stoenoiu, Alexandre Persu, Rosario Pascarella

**Affiliations:** 1 Neurology Unit, Stroke Unit, Azienda Unità Sanitaria Locale-IRCCS di Reggio Emilia, Viale Risorgimento 80, 42123 Reggio Emilia, Italy; 2 Department of Internal Medicine, Rheumatology, Institut de Recherche Expérimentale et Clinique, Cliniques Universitaires Saint-Luc, Université Catholique de Louvain, 1200 Brussels, Belgium; maria.stoenoiu@saintluc.uclouvain.be; 3 Division of Cardiology, Cliniques Universitaires Saint-Luc, Université Catholique de Louvain, 1200 Brussels, Belgium; 4 Pole of Cardiovascular Research, Institut de Recherche Expérimentale Et Clinique, Université Catholique de Louvain, 1200 Brussels, Belgium; 5 Neuroradiology Unit, Ospedale Santa Maria della Misericordia, AULSS 5 Polesana, 45100 Rovigo, Italy; rosario.pascarella@aulss5.veneto.it

**Keywords:** intracranial aneurysms, subarachnoid hemorrhage, fibromuscular dysplasia, dissection, endovascular, neurosurgical treatment, angiography

## Abstract

**Background**: Fibromuscular dysplasia (FMD) is a non-inflammatory vascular disorder that affects medium and large arteries, with a notable association with intracranial aneurysms (IAs). This review aims to assess the prevalence, characteristics, and implications of IAs in patients with FMD, highlighting gaps in current knowledge and the need for further research. **Methods**: A comprehensive literature search was conducted on PubMed using keywords related to FMD and intracranial aneurysms. The search focused on studies published over the last 28 years, identifying relevant data on the prevalence and morphological features of IAs in FMD patients. Due to the limited quality and availability of information, a narrative review format was adopted to synthesize findings. **Results**: The review found that the prevalence of IAs in FMD patients is significantly higher than in the general population, with estimates varying widely (4.7–21.7%). The majority of patients identified with IAs were female, and the age range of affected individuals varied significantly. Key risk factors for aneurysm formation included hypertension, smoking, and the presence of multifocal or multisite FMD. Notably, the study indicated that routine screening for IAs in FMD patients has to be weighted with the relatively low prevalence of asymptomatic IAs and the risk-to-benefit ratio of treatment in older patients. **Conclusions**: The association between FMD and intracranial aneurysms is significant, and timely detection of these aneurysms may allow preventing subarachnoid hemorrhage, whose fatality rate is high. Identification of subgroups where the screening may be cost-effective, also considering the impact of the awareness to have an IA without treatment proposal, is warranted. Further research is essential to clarify the relationship between FMD and IAs, optimize screening protocols, and improve outcomes for affected patients. The findings underscore the importance of ongoing registries to enhance understanding of the natural history and treatment of IAs in the context of FMD.

## 1. Introduction

Fibromuscular dysplasia (FMD) is a non-inflammatory and non-atherosclerotic vasculopathy which may involve medium- and large-caliber arteries virtually in every vascular bed in the body [1,2]. The most frequently involved arteries are renal arteries and extracranial cerebrovascular arteries. Usually, more arterial beds are involved. FMD is morphologically differentiated into multifocal and focal FMD, characterized by the classical string-of-beads appearance and by a focal short or long stenosis, respectively [1,2]. Nevertheless, this morphological classification is practical but oversimplified, flattening the wide range of imaging phenotypes previously described and including atypical subtypes (not only carotid web) [3,4,5,6]. Therefore, the ongoing registries are of paramount importance to better identify the different clinical and radiological phenotypes and to assess their natural history. Besides the arterial lesions considered typical for FMD diagnosis, several other arterial conditions are identified in patients with FMD with a higher prevalence than in general population, and in particular dissections and aneurysms. Intracranial aneurysms were previously considered present in up to a half of FMD patients [7,8,9], but in the last 30 years, the development on noninvasive techniques for vascular imaging and the increased proportion of asymptomatic or incidental FMD diagnoses, reduced this rate to 10–20% [1,2], mainly unruptured intracranial aneurysms (UIAs). Unfortunately, the published data, including those reported in the international registries, did not deeply address the morphological features and the natural history of ruptured and unruptured aneurysms in patients with FMD and several issues remain unsolved.

Accordingly, in this review we aimed to identify and extract information from the literature in order to better characterize intracranial aneurysms in patients with FMD and to identify the missing pieces of the puzzle for future research.

## 2. Materials and Methods

The authors performed an extensive research on PubMed using several combination of the following terms: “intracranial aneurysm*”, “FMD”, “fibromuscular dysplasia” and “spontaneous subarachnoid hemorrhage” without time limits. Considering that a single relatively old (1998) meta-analysis about the prevalence of intracranial aneurysms was identified [10] and, otherwise, only isolated reports encompassing the last 28 years were retrieved, the known published registries of FMD were searched for information about intracranial aneurysm. Overall, both the quality of information regarding intracranial aneurysms and its reproducibility appear substantially limited, making the initially planned approach of a systematic literature review impractical. We therefore used the identified data for a narrative review aimed at defining the following issues: (1) proportion of ruptured and unruptured intracranial aneurysms in patients with FMD; (2) proportion of FMD diagnoses in patients with spontaneous subarachnoid hemorrhage; (3) morphological features of aneurysms in FMD patients; (4) impact of FMD on management of IA.

## 3. Results

### 3.1. Proportion of Ruptured and Unruptured Intracranial Aneurysms in Patients with FMD

Starting from the single, available meta-analysis on this topic, the information about the prevalence of intracranial aneurysms in FMD patients changed across the decades [10]. In a significant reassessment of the prevalence of cerebral saccular aneurysms among patients with FMD, in 1998 Cloft et al. [10] conducted a thorough study that provided noteworthy insights into this vascular condition. Their research, which encompassed a meta-analysis of existing literature and an evaluation of their own patient cohort, aimed to clarify the actual relationship between FMD and the occurrence of aneurysms. The meta-analysis included data from 17 published studies, overall involving 498 patients diagnosed with internal carotid artery (ICA) and/or vertebral artery (VA) FMD. Among these patients, a striking proportion of 22% were found to have an aneurysm, 76 of whom with ruptured aneurysms, symptomatic for subarachnoid hemorrhage (SAH), or unruptured symptomatic aneurysms, requiring further investigation. However, when the authors focused on incidental and asymptomatic cases, the calculated prevalence dropped to approximately 7.6%, highlighting a significant disparity compared to previous reports suggesting figures as high as 51% [11,12,13,14,15]. To bolster their findings, Cloft et al. [10] also retrospectively analyzed a cohort of 117 patients from their institution, showing that 24% of these individuals harbored an intracranial aneurysm, with 22% of them exhibiting symptoms that prompted angiography. Notably, after excluding symptomatic cases, the prevalence of incidental aneurysms was recorded at 6.3 ± 4.9%. When combined with the data from the meta-analysis, the overall prevalence of incidental, asymptomatic aneurysms in patients with FMD was established at 7.3 ± 2.2%. This figure aligns closely with estimates for the general population, suggesting no statistically significant difference between the two groups [10]. The study further illuminated the gender dynamics within this patient population, revealing that a substantial majority of patients—85%—were female. This demographic detail is crucial, as it underscores the higher risk of aneurysms typically faced by women. The age range of patients varied widely, from 4 to 83 years, emphasizing the diverse nature of subjects affected by FMD. An updated prevalence of incidentally discovered IAs in the general population was lower than the one reported as comparison by old studies [10] and ranging from 2% to 3.2%, with a higher prevalence in women, especially those over 50. While studies vary, female sex predominance is a consistent risk factor, with some research finding the prevalence of UIAs in women to be nearly twice that of men [16,17].

This study [10] identified a distribution of aneurysms across various locations: 9 in the anterior communicating artery, 7 in the posterior communicating artery, and n in the ophthalmic artery, ICA, middle cerebral artery, and basilar tip. The rare of multiple aneurysms was 21% of patients with FMD and IAs. The authors emphasized the importance of addressing selection bias in previous studies that reported higher prevalence rates of aneurysms in FMD patients. Many studies indeed included patients undergoing imaging due to symptomatic presentations, which likely inflated prevalence estimates. Cloft et al. [10] argue that valid comparisons of aneurysm prevalence should rely on data from patients not presenting with symptoms related to aneurysms. Their study suggests that the previously reported prevalence rates of 21% to 51% are likely overestimated and that the actual prevalence of incidental, asymptomatic aneurysms in FMD patients is closer to 7%. The high percentage of women among FMD patients may contribute to a perceived increased prevalence of aneurysms, as women are generally at higher risk for such vascular conditions.

The findings raise questions regarding the necessity of routine screening for aneurysms in FMD patients. Given the relatively low prevalence of asymptomatic aneurysms found in this study, routine screening may not be justified. The authors recommend that future studies consider the broader implications of FMD as a systemic condition and explore screening protocols for patients with FMD affecting arteries outside the ICA and VA.

Cloft et al. [10] also call for more comprehensive studies aiming to clarify the relationship between FMD and cerebral aneurysms, as well as to determine the utility of specific screening techniques (like magnetic resonance angiography or computed tomography angiography) for detecting aneurysms in asymptomatic FMD patients.

Notably, Clofts et al. [10] considered solely the prevalence of saccular intracranial aneurysms, but information coming from international FMD registries is less precise about the shape of the aneurysms considered. Therefore, we have to assume that every intracranial aneurysm was included, irrespectively from their subtype (e.g., saccular, fusiform or dissecting). Data form the United States of America FMD registry [18] are mainly based on non-neurological centers and show us quite a different picture. The study involved a comprehensive analysis of the U.S. Registry for FMD, which included 921 patients. Among these, 200 patients (21.7%) were found to have an aneurysm (most frequent location: carotid and renal arteries), while 237 patients (25.7%) experienced a dissection, with 53 patients (5.8%) presenting with both conditions. Notably, the majority of patients in this cohort were women, constituting 93.5% of the total population, which aligns with the general demographic trend observed in FMD cases [19]. IAs were identified in 43 patients, representing 21.5% of patients with aneurysms and about 4.7% of the overall cohort. This prevalence is significantly higher than the estimated 2% to 3.2% prevalence of cerebral aneurysms in the general population [16,17,20]. Moreover, in patients who presented with SAH, the prevalence of cerebral aneurysms soared to as high as 50% [10,18]. The study emphasizes the importance of recognizing the potential for IA formation in FMD patients, as these aneurysms can lead to life-threatening complications such as SAH. In fact, 8.4% of patients with aneurysms had a prior history of SAH, indicating a significant risk associated with these vascular abnormalities. Furthermore, the study reported that 63 out of 200 patients (31.5%) with identified aneurysms underwent therapeutic interventions aimed at repairing these vascular lesions. Among these, endovascular therapy was the most commonly performed procedure, with 36 patients (57.1%) receiving this treatment, while 26 patients (41.3%) underwent surgical interventions. Specifically, 16 of the 44 patients with IAs had procedures aimed at preventing aneurysm rupture, underscoring the critical need for timely intervention in this high-risk group. The findings from Kadian-Dodov et al. [18] highlight the high prevalence of IAs in FMD patients and the necessity for routine imaging—either computed tomographic angiography (CTA) or magnetic resonance angiography (MRA)—to identify these potentially dangerous conditions early. Given the increased risk of SAH and other complications, the authors recommend that all patients diagnosed with FMD undergo comprehensive imaging from head to pelvis to assess for aneurysms and dissections [13]. In summary, the study provides suggestive evidence of the significant relationship between FMD and the development of IAs. The information presented emphasizes the need for awareness and proactive management strategies to mitigate the risks associated with these vascular anomalies in affected patients.

In the European FMD registry [2], the analysis of 1022 patients enrolled in the FMD Registry revealed that the majority (82%) were women, with an average age of diagnosis of 46 years. Hypertension was the most prevalent symptom, affecting 86% of patients. Notably, 21.6% of patients had at least one aneurysm, and 5.6% had experienced a dissection. The presence of aneurysms was significantly associated with the multifocal and multivessel types of FMD, which were present in 72% and 57% of patients, respectively. The prevalence of aneurysms in patients with FMD was confirmed to be approximately 21.6%, indicating a heightened risk when compared to the general population, but without a separated analysis of the involved vascular beds.

Multivariate analysis identified key predictors of aneurysms in FMD patients, including:

- Multivessel FMD (OR 3.99, 95% CI [2.89–5.57], *p* < 0.001)
- Multifocal FMD (OR 1.91, 95% CI [1.26–2.98], *p* = 0.003)

While the overall prevalence of aneurysms did not significantly differ between men (18.8%) and women (22.3%), men exhibited a higher frequency of arterial dissections (14% vs. 3.6% in women).

More information about the issue of associated aneurysms, including IAs, was present in the cohorts enrolled in the ARCADIA [21] and in the ARCADIA-POL [22] studies, having the advantage of a mandatory whole body vascular screening in the proposed patients with FMD with a preplanned follow-up.

In Table 1, a summary of the features of patients with FMD and intracranial aneurysms from the three described cohorts has been provided.

### 3.2. Prevalence of FMD in Patients with SAH

SAH is most often caused by a ruptured IA (85% of cases), with hypertension, cigarette smoking, and a family history being major risk factors. It accounts for about 5% of all strokes, with a worldwide incidence of approximately 9.1 per 100,000 person-years, though this can vary significantly by region and has generally been declining [23]. SAH is more common in women than men, and while incidence increases with age, approximately 50% of cases occur in people under 55 years old [23].

The global incidence of aneurysmal SAH in a systematic review and meta-analysis from 2019 was 7.9 per 100,000 person-years. This analysis examined 75 studies from 32 countries, reporting 3427 cases in the European population, 3581 in the Asian population, 513 in Australia and New Zealand, 429 in North America, 2016 in South America, and 20 in Nigeria. Temporal trends indicated that the incidence in 2010 was 6.1 per 100,000 person-years, a reduction from 10.2 per 100,000 person-years in 1980. Notably, there is considerable geographic variation in the incidence of aneurysmal SAH, with rates ranging from 6.9 per 100,000 person-years in North America to 28 per 100,000 person-years in Japan. In Asian countries, excluding Japan, the reported incidence was 3.7 per 100,000 person-years. A Swiss national inpatient database from 2009 to 2014 recorded an incidence of 3.7 per 100,000 person-years [24]. The average age for aneurysmal SAH is between 50 and 55 years [25,26], but children and the elderly can also be affected [23]. African Americans appear to be at a higher risk compared to Caucasian Americans [27]. Women have a higher incidence of aneurysmal SAH, likely related to hormonal status [23]. A meta-analysis of nine prospective studies assessing risk factors and rupture rates of 9940 intracranial aneurysms found that the rupture rate was higher for women than for men (1% vs. 0.7%, hazard ratio 1.43, 95% CI 1.1–1.9) [28]. This increased rupture rate for women remained significant even after adjusting for other associated risk factors, including smoking (current or past), hypertension, previous aneurysms, and family history of aneurysms. Risk factors for aneurysmal SAH (or IA rupture) are related to both the anatomical characteristics of the aneurysm and patient-related factors. Besides female sex, the most consistent risk factors are hypertension, cigarette smoking, and family history of aneurysms [23].

FMD is a predisposing factor for SAH due to an increased risk of cerebral aneurysms. In patients with FMD who have already experienced SAH, the prevalence of associated cerebral aneurysms can be very high, potentially reaching 50% or more in some series. However, no studies directly addressed the rate of FMD in patients presenting with SAH or with aneurysmal SAH.

### 3.3. Features of Intracranial Aneurysms

In the context of FMD, the presence of IAs poses a critical concern due to their potential for rupture, which can lead to severe consequences such as SAH.

Regarding the location and size of intracranial aneurysms in relation to rupture risk, two large prospective studies have reported on the natural history of unruptured intracranial aneurysms: the International Study of Unruptured Intracranial Aneurysms (ISUIA) [29,30], which prospectively evaluated 1692 patients with 2686 unruptured and untreated aneurysms in the USA, Canada, and Europe, and the Unruptured Cerebral Aneurysms Study (UCAS) [31], a Japanese cohort of 6697 aneurysms in 5720 patients. Both studies established a clear relationship between both the location and size of the aneurysm and the risk of rupture. The PHASES score, developed from a pooled analysis of six prospective cohort studies, incorporated age, hypertension, maximum aneurysm diameter, previous SAH history, and aneurysm location as key predictors of rupture risk, providing a useful tool for individualized treatment strategies [32]. Additionally, the ISUIA and UCAS studies confirmed previous findings regarding the lower rupture rates of smaller aneurysms. The threshold diameter in both studies for defining a low rupture risk was set at 7 mm, with a progressive increase in rupture risk as size exceeds this threshold. In the ISUIA, for anterior circulation aneurysms, the 5-year rupture rates were 2.6% for aneurysms ranging from 7–12 mm, 14.5% for those from 13–24 mm, and 40% for those > 25 mm. Another prospective cohort study followed 374 patients with 448 aneurysms < 5 mm, finding an average annual rupture rate of 0.54% overall, 0.34% for single aneurysms, and 0.95% for multiple aneurysms [33]. In this cohort, the risk of aneurysm rupture was also higher in patients aged < 50 years and in those with aneurysms > 4 mm. The hazard ratios reported in the UCAS, using 3–4 mm aneurysms as a reference, were 3.3 for aneurysms in the 7–9 mm range, 9.1 for those in the 10–24 mm range, and 76.3 for those ≥ 25 mm. Moreover, the increase in size of aneurysms is more likely to occur in larger aneurysms compared to smaller ones [34]. In fact, in the aforementioned study, among 165 patients with 191 UIAs, the growth frequency over 47 months was 7%, 25%, and 83% for aneurysms < 8 mm, 8–12 mm, and >13 mm, respectively. Aneurysms of the internal carotid artery (siphon) and the basilar artery had a greater likelihood of growth compared to those in other locations [35].

**Figure 1:**
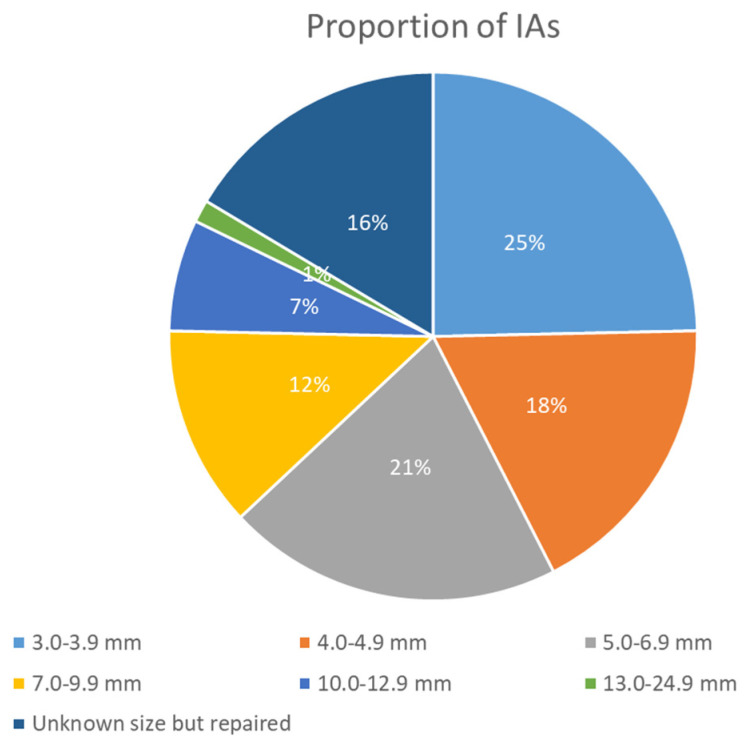
Graph of IAs size reported in USA FMD registry [36].

An article published in 2017 provided more details about the features of IAs in patients enrolled in the US Registry for FMD [36]. Specifically, out of 669 women who underwent intracranial imaging, 12.9% were found to have at least one intracranial aneurysm, a figure that surpasses general population estimates, although this may partly reflect a selection bias. Notably, a considerable percentage of these women had multiple aneurysms, with 30.2% exhibiting more than one intracranial aneurysm. The prevalence of IAs varied only slightly depending on the location of FMD; specifically, 11.9% of patients with renal involvement and 13.7% with cervical involvement had intracranial aneurysms. This suggests that the location of FMD does not significantly influence the prevalence of intracranial aneurysms. Among the 86 women identified with intracranial aneurysms, various characteristics were noted that underscore the potential risk factors and clinical implications:

1. **Size of Aneurysms:** 56/128 (43.2%) aneurysms were ≥5 mm, i.e., at high risk of rupture, illustrating a higher risk profile compared to general population findings. The sizes were categorized as in Figure 1.

2. **Location of Aneurysms:** The distribution of IAs was notable, with a significant number located in areas typically associated with higher risks of rupture. A substantial 41% of the identified intracranial aneurysms were located in the intradural segments of the ICA, which is considered a high-risk area, along with 12% in the posterior communicating artery and 9% in posterior circulation arteries.
3. **Multiplicity:** Many patients presented with multiple IAs, indicating a possible underlying susceptibility linked to the pathophysiology of FMD. The median number of aneurysms per patient was 1, but some had as many as 8 intracranial aneurysms.

The study revealed a strong association between a history of smoking and the presence of IAs, with 53.8% of patients with IAs being smokers compared to 28.9% among those without. This correlation underscores the need for heightened awareness and potential screening for IAs in smokers diagnosed with FMD.

In the other reported studies including patients with FMD and IAs [2,10], much less details were provided about the size and morphological features of IAs.

### 3.4. Treatment Issues

The clinical implications of these findings are important, as intracranial aneurysms pose a risk of rupture, leading to SAH, a condition with high morbidity and mortality rates. The mortality rate among patients experiencing SAH due to intracranial aneurysm rupture in the general population is approximately 50%, indicating a critical need for preventive measures and early detection [37,38].

Current guidelines recommend that all patients with FMD undergo screening for IAs, particularly due to the higher prevalence observed in this population [1]. Non-invasive imaging techniques such as MRA or CTA can be employed to identify IAs before rupture occurs.

Despite the potential benefits of screening, the risks associated with intervention must be balanced against the likelihood of aneurysm rupture. The decision to treat an IA—whether through surgical or endovascular means—depends on various factors, including the aneurysm’s size, location, and the patient’s overall health status. The impact of FMD status on decisions for aneurysms treatment and prognosis is not yet well established.

Bender et al. [39] explored the unique challenges and outcomes associated with treating cerebral aneurysms in patients diagnosed with FMD. FMD is associated with an increased prevalence of cerebral aneurysms and dissections, particularly in the mid-cervical carotid and vertebral arteries. This condition often discourages clinicians from pursuing elective endovascular treatments due to concerns about safety and efficacy. The study involved a review of a prospectively maintained database at a tertiary medical center, which included 1025 patients undergoing intracranial embolization procedures. Among these patients, only 31 (3.0%) were identified as having cerebrovascular FMD. Collectively, they underwent 43 embolization procedures, with 27 of these procedures performed in vessels affected by FMD. Notably, the majority, 30/31 (96.8%), of these patients were women, with a mean age of 62 years, and the most common FMD subtype observed was the “string-of-beads” type, affecting 90% of the cases. The ICA was more frequently involved than the VA, with overlapping involvement in both the anterior and posterior circulation. The average size of treated aneurysms was reported as 7.1 mm, with a predominance of saccular morphology observed in 93% of the cases. Most aneurysms (87%) were discovered incidentally, and a notable 19% of patients had incidental vessel dissections. The endovascular treatment techniques utilized included flow diversion, coiling, stent-coiling, and intrasaccular flow disruption. Among these, flow diversion was the most common treatment, accounting for 67% of the procedures. Remarkably, all but one procedure (98%) were successful, with no major complications reported, although one patient did experience a transient ischemic attack during recovery. Follow-up angiography was conducted in 88% of the cases, showing no evidence of disease progression after treatment, further supporting the safety of elective endovascular interventions in this patient population. However, the study has several limitations. One significant concern is the reliance on angiographic definitions of FMD without corresponding histopathological confirmation. The study’s retrospective nature means that it may not capture the full complexity of FMD’s impact on aneurysm treatment outcomes. Additionally, the authors noted that their center’s status as a referral institution might have introduced biases in the patient selection process, and the incomplete follow-up in some cases limits the ability to draw definitive conclusions about long-term outcomes.

Finally, Bender et al. [39] present compelling evidence that elective endovascular treatment of IAs can be performed safely in patients with FMD. They provide valuable insights into the management of cerebral aneurysms in this unique patient population, emphasizing the need for careful evaluation and reporting in future studies to improve treatment protocols and patient outcomes. Nevertheless, as indicated by the authors, further research is necessary to fully understand the implications of FMD on aneurysm management and to establish standardized treatment guidelines.

## 4. Discussion

**Figure 2:**
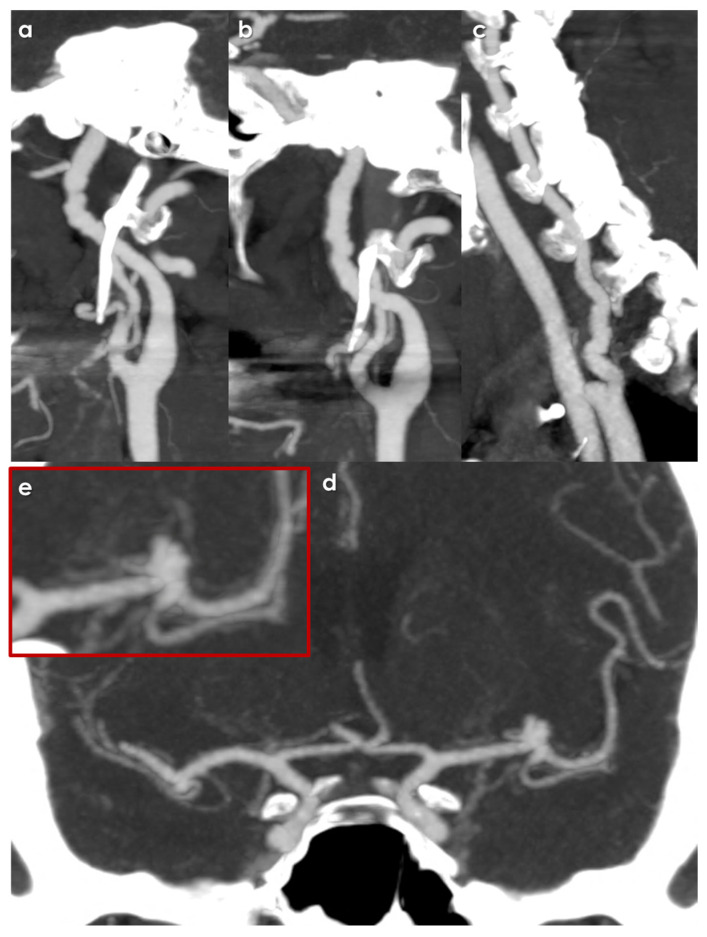
CT Angiography of the cervical and intracranial arteries performed as screening for FMD in a patient who underwent a spontaneous coronary artery dissection (renal and visceral arteries were also explored without abnormalities). All images are Multiplanar Reconstructed and processed with Maximum Intensity Projection protocol (MIP/MPR). Panel (**a**) shows the right extracranial ICA with the multifocal pattern (string of beads), as for left ICA (panel (**b**)) and left V1 VA (panel (**c**)). In panel (**d**) and (**e**, magnified view), a left MCA bifurcation aneurysm is evident (well detailed using catheter angiography, showed in Figure 2).

**Figure 3:**
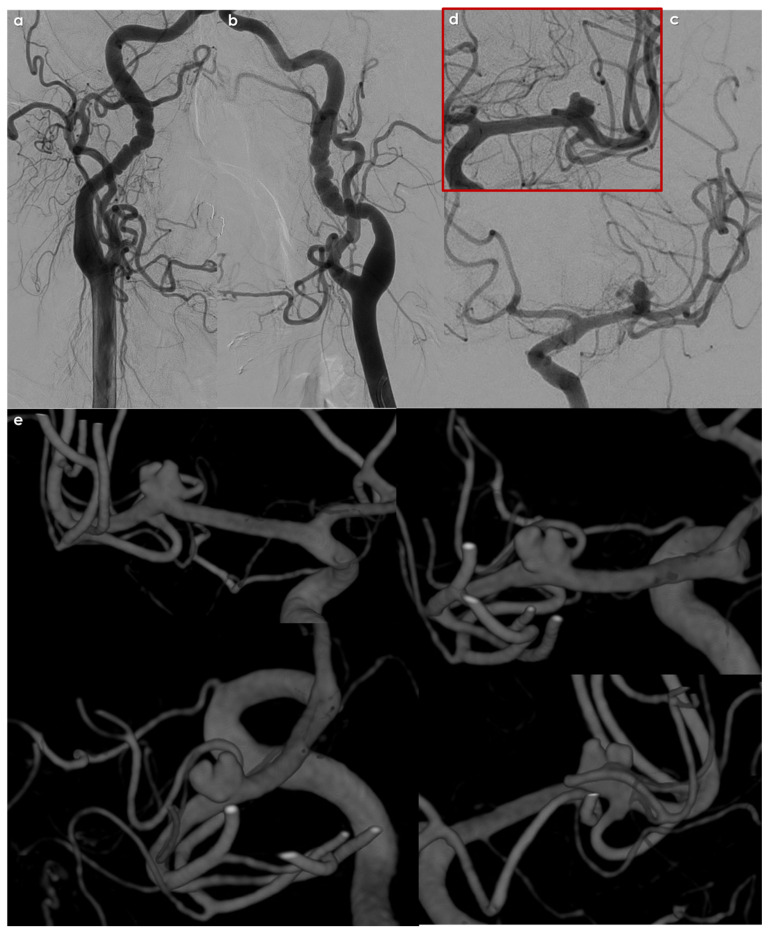
Digital subtraction angiography (DSA) in the same patient as in Figure 1. Multifocal FMD is evident on both the right (panel (**a**)) and the left (panel (**b**)) ICA. In (panel (**c**)), the left MCA bifurcation saccular aneurysm is imaged (and magnified in panel (**d**)) with an irregular surface and the appearance of two blebs. In panel (**e**), multiple views of the 3D rotational angiography with volume rendering technique are well evident, as well as the wide neck with an MCA branch originating from the neck. The patient was candidate to a surgical treatment.

From the presented data, it is quite clear that, to date, description of intracranial aneurysms in FMD patients is often incomplete and inconsistent. Another concern is the lack of central reading in the studies in question, which, in a less experienced context, could have led to a potential underestimation of the prevalence of aneurysms, particularly in the <5 mm diameter category. Furthermore, aspects of follow-up and risk of SAH in the cohort of patients with FMD and UIAs have not been fully explored. However, it may be hypothesized that spontaneous SAH may in some cases be associated with non-aneurysmal pathology, particularly intracranial dissection. An example of an intracranial aneurysms incidentally discovered during screening for FMD is shown in Figure 2 and Figure 3.

Another important issue is the potential influence of ethnic origin, particularly in view of the known higher prevalence of intracranial aneurysms in the Asian population. A recent systematic review and meta-analysis [40], performed in Asian patients with FMD, highlighted relevant aspects related to aneurysms. Among the 1254 Asian participants included in the meta-analysis, aneurysms related to FMD were present in 13.5% of the cases (95% CI: 7.8–20.4%). This prevalence indicates a significant association between FMD and of aneurysms within this population, though lower than in previous reports mostly involving Caucasians (22.7% in the US and 21.6% in European studies). Notably, the analysis revealed that the prevalence of FMD-related aneurysms was higher in Chinese patients (15.5%) compared to Japanese (3.2%) and Taiwanese (5.3%) patients. The study’s findings also suggest that Asian patients with FMD tend to present with a distinct clinical profile compared to Caucasians. In particular, Asian patients were generally younger at diagnosis (mean age of 28.6 years), with a higher prevalence of focal rather than multifocal FMD. However, this systematic review may not reflect the whole range of FMD presentation in Asian patients, as FMD was usually identified in the context of a work-up for arterial hypertension and vascular beds other than the renal arteries were not systematically screened.

The association between intracranial aneurysms and FMD is of particular clinical relevance, as these aneurysms can lead to severe complications, including hemorrhagic strokes. The data underscore the necessity for heightened awareness and screening for intracranial aneurysms among patients diagnosed with FMD, particularly in Asian patients, where genetic and environmental factors may contribute to variations in disease presentation. The implications of these findings are significant, highlighting the need for further research to explore the underlying mechanisms linking FMD and intracranial aneurysms. Genetic studies and investigations into environmental risk factors may provide deeper insights into how these conditions coexist and could inform tailored management strategies for affected individuals.

The characteristics of the FMD are barely reported in most studies, preventing us from confidently defining a differentiated profile of patients with FMD and intracranial aneurysm. For example, only European registry data [2,21,22] provide information on the FMD subtype (focal vs. multifocal) and its association with the presence of aneurysms, but the single mention of intracranial bleeding as a proxy for IAs is SAH. This also represents an area requiring more careful exploration, particularly considering the possibility of a more detailed phenotypic classification of neurovascular FMD. Furthermore, the available characterization of UIAs in FMD registries does not allow to assess individual risk based on validated scores such as PHASES.

**Figure 4:**
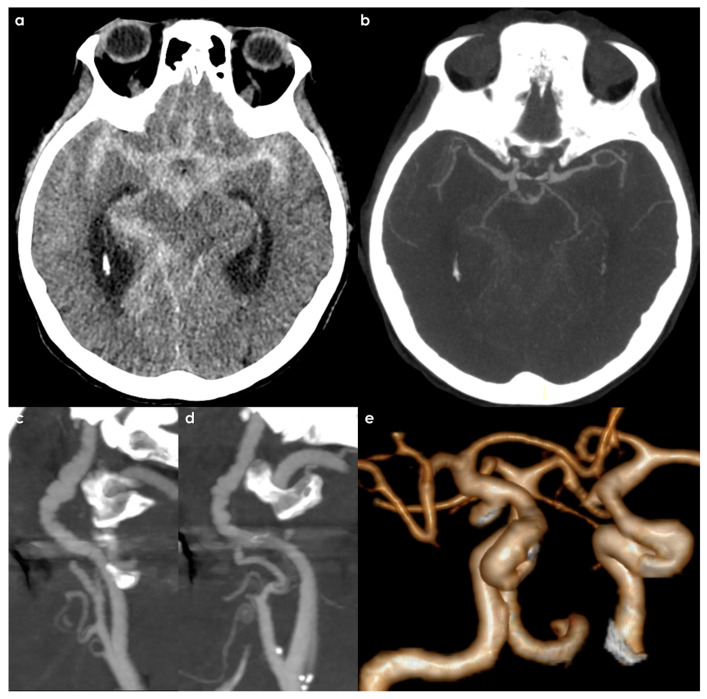
CT and CTA of a patient admitted for the abrupt onset of (thunderclap) headache and progressive decrease in consciousness. The non-contrast brain CT (panel (**a**)) showed a massive SAH (Fisher grade 4). The CTA (panel (**b**)) highlighted the presence of a right ICA saccular aneurysm in the communicating segment (MIP/MPR images). In the same investigation, both right (panel (**c**)) and left (panel (**d**)) ICA have a multifocal FMD pattern. A volume rendering reconstruction of the intracranial arteries (panel (**e**)) gives more details about the morphology and surface of the aneurysm.

**Figure 5:**
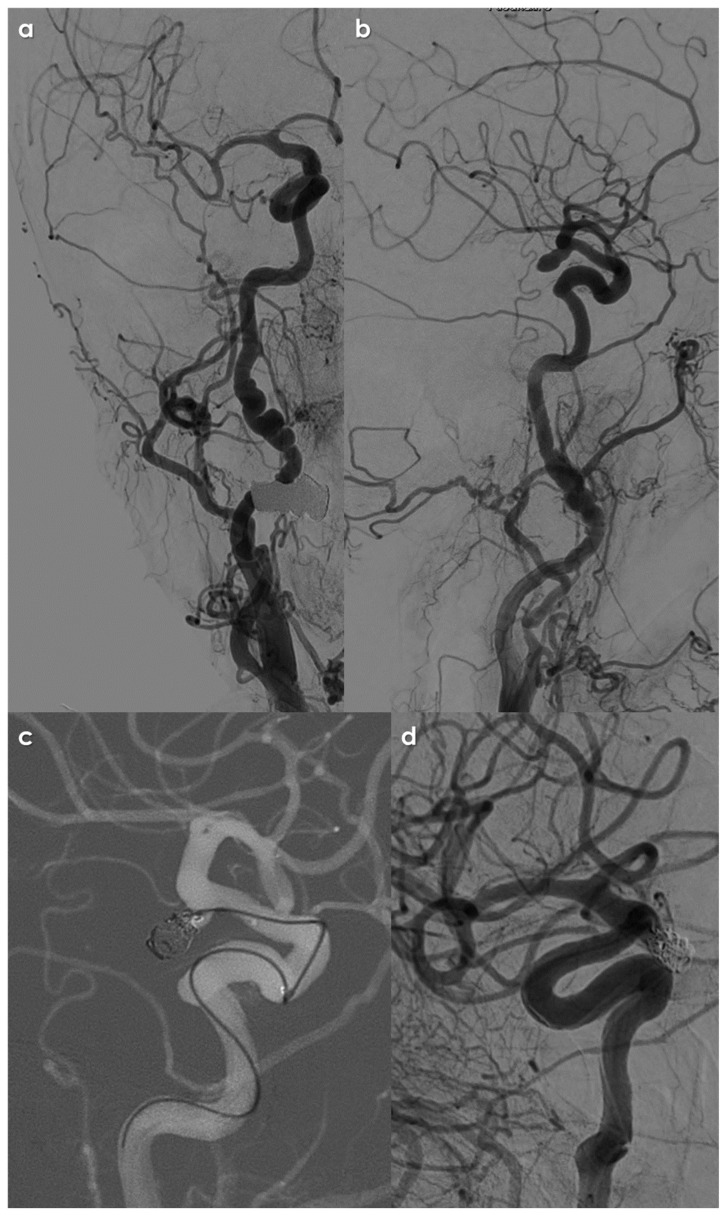
DSA and embolization procedure in the same patient as in Figure 3. Following left (panel (**a**)) and right (panel (**b**)) common carotid artery injection, the multifocal FMD pattern is well detailed on both ICAs. In panel (**b**) (lateral view) the right ICA aneurysm is also highlighted. Panel (**c**) shows the final steps of coiling procedure, before detaching the last coils and, in panel (**d**), the angiographic check after the procedure confirmed the complete exclusion of the aneurysmal sac from the circle of Willis.

The situation of patients with spontaneous SAH, whether aneurysmal or not, is almost completely unexplored. Indeed, the prevalence of FMD in these patients is unknown. This issue may be a relevant research question because, partially due to the poor prognosis of some subsets of patients, FMD may be easily underdiagnosed. An example is proposed in Figure 4 and Figure 5.

Finally, beyond individual follow-up recommendations, the natural history of aneurysmal disease in patients with FMD is unknown, nor is it possible to define whether there is an increased risk of de novo aneurysms during follow-up. A significant proportion of these patients also have associated vascular risk factors, such as hypertension and smoking, and these factors, and their management, may also impact the progression of the disease.

A strong female predominance is observed in patients with IAs, as both in FMD registries and in the general population. The presence of aneurysms appears to be a characteristic feature of FMD, even in patients with spontaneous carotid or vertebral dissection [41].

Finally, the treatment (conservative, endovascular, surgical) of aneurysms, particularly UIAs, in the FMD patient population has not received much attention, partly reflecting a theoretical concern regarding the tentative higher risk of iatrogenic dissection during arterial catheterization in these patients. A further consideration is that the overall quality of studies reporting the outcomes of treatments for intracranial aneurysms is low-to-moderate. In a comprehensive systematic review and meta-analysis conducted by Volovici et al. [42], the authors aimed to evaluate the safety, effectiveness, and durability of various treatments for IAs. This investigation arose from the need to rigorously assess new medical devices and procedures before their implementation in clinical practice. The review screened a staggering 12,954 studies, ultimately including 1356, which encompassed a total of 410,993 treated patients. Of these, 261,675 patients underwent endovascular treatment, while 149,300 were treated microsurgically. The analysis revealed that approximately 80% of the included studies reported positive conclusions regarding safety or effectiveness. However, the majority of these studies were single-group and retrospective in nature, lacking the robust methodological rigor often required for meaningful conclusions. For instance, 1060 intervention groups assessed safety, with 821 (77%) classified as safe, 235 (22%) as uncertain, and just 4 (0.4%) marked as unsafe. Interestingly, the in-hospital mortality rates did not significantly differ between studies with positive, uncertain, or negative conclusions, highlighting a concerning trend where claims of safety may not be substantiated by rigorous evidence. When examining effectiveness, the review found that of the 1025 intervention groups evaluated, 826 (81%) were deemed effective. However, the rates of complete occlusion at discharge showed no substantial differences irrespective of the various conclusions drawn by the studies. For example, the complete occlusion rates for studies with positive conclusions (61.4%) were nearly identical to those with uncertain conclusions (62.0%) and only slightly more favorable than those labeled as negative (51.8%). This lack of distinction raises questions about the reliability of these claims, suggesting that many studies may not have adequately controlled for variables associated with successful outcomes.

Durability, another critical factor in assessing IA treatments, was evaluated across 164 intervention groups. The findings indicated that 58% were considered durable, yet, once again, the median rates of complete occlusion at final follow-up did not significantly vary between groups with positive (83.1%), uncertain (76.9%), or negative conclusions (70.3%). This lack of differentiation suggests that both the definitions and thresholds for determining durability were inadequately addressed in most studies, as only a handful provided clear criteria. A significant limitation of the reviewed literature was the high incidence of improper reporting, with 40% of studies exhibiting flaws in how outcomes were presented. Furthermore, definitions for safety, effectiveness, and durability were alarmingly absent in most papers, with only 2% of studies offering any form of definition. This absence undermines the credibility of the findings and indicates a potential public health risk, as unsubstantiated claims may lead to the adoption of inferior treatment methods. Moreover, the authors noted that the study design of many included works was suboptimal, as single-group studies lacked the necessary comparative benchmarks to evaluate the true efficacy of new treatments against current standards. This finding is particularly troubling as it suggests a systemic issue in the field, where claims of safety and effectiveness may be overstated due to a lack of appropriate controls. Only 0.002% of studies included incidence thresholds for safety or effectiveness, reflecting a concerning trend of vague reporting practices that ultimately hinder the advancement of knowledge in the field.

As previously reported, the limitations of the available studies do not allow us to define a dedicated approach to the management of UIAs in patients with FMD, nor do they provide reliable information on the risk/benefit ratio of the different possible treatment strategies (surgical, endovascular, wait and see). In particular, when addressing this issue from the FMD perspective, i.e., from the point of view of the information provided by cohort studies and observational registries, the following biases are evident: (1) the registries and their respective CRFs were not designed to obtain detailed information on neurovascular aspects; the consequence of this approach is that intracranial aneurysms are recorded as present or absent, but often without separating them from aneurysms of other vascular districts and from dissections; (2) no details are given on the elements that influenced the therapeutic choice with regard to UIAs, but the possibility of treatment (surgical or endovascular) is simply recorded, but without specifying the modalities and technique; (3) very little information is given on the morphological characteristics of UIAs and in some cases neither their location nor size is indicated, which makes it difficult to apply, even retrospectively, scales that allow for estimating the risk of rupture; (4) the methods of investigation of UIAs are not detailed, which may be relevant for defining an individualized risk of rupture and therefore guiding the choice of treatment (e.g., CTA vs. MRA or DSA; Vessel Wall Imaging-MR) [43]; (5) None of the available studies used a centralized reading of neuroradiological and radiological investigations by professionals experienced in FMD, but what was reported derives from the local reading of individual centers, presumably by operators with different experience. Approaching this issue from the point of view of the incidental finding of a UIA in the population, as evidence of the lack in clinical practice of a dedicated investigation pathway to identify the presence of an underlying systemic arteriopathy, there are no studies that have considered systemic screening for FMD in patients with recurrent UIAs, neither in the neuroradiological nor in the neurosurgical field. The same considerations are valid, in both fields, for patients with SAH due to aneurysm rupture. FMD is not a genetic disease (and in particular, it is not a monogenic disease), so even recent studies on the genetic background of intracranial aneurysms do not include or consider it [44]. Finally, registry data may be somewhat influenced by the setting of the participating centers, very few of which are neurovascular, compared to the majority with a cardiology background and dedicated to the management of arterial hypertension. Many patients with incidental diagnoses are likely still lost and do not contribute significantly to a modern and comprehensive definition of the characteristics and history of the disease, including potentially associated vascular lesions (aneurysms and dissections first). Summarizing, in most of the reported studies there is a detection bias, affecting the reported rate of patients with IAs. In fact, the rate of aneurysms (in every location) per registry is available, but not the number of aneurysms per screened vascular beds. On the other hand, studies in patients with multiple IAs usually do not include a screening of other arterial beds, decreasing the probability to diagnose underlying FMD [45]. Similarly, familial screening of patients with IAs and familial, recurrence is usually focused on the intracranial arteries.

Therefore, a two-pronged approach would be necessary: analyzing registry data with a dedicated multidisciplinary neurovascular approach, starting with defining the characteristics of IAs and moving on to an individualized assessment of the risk of rupture and defining the treatment strategy with the best risk/benefit ratio. Furthermore, it seems appropriate to recommend systematic screening for FMD in patients presenting with SAH from aneurysmal rupture and UIAs.

## 5. Conclusions

The prevalence of intracranial aneurysms in patients (mainly women) with FMD is notably higher than in the general population, emphasizing the importance of screening and monitoring. The characteristics of the intracranial aneurysms found in this population, including their size, location, and multiplicity, suggest a heightened risk for complications. With a notable prevalence of aneurysms, particularly in patients with multifocal and multivessel FMD, targeted screening and individualized management strategies are essential for improving outcomes. As previously pointed out, several questions remain without answers and there is wide room for a future targeted research in this field, optimizing the data from the ongoing registries.

## Figures and Tables

**Table 1:** Summary of intracranial aneurysms prevalence and features in FMD patients.

Category	Clefts et al. [10]	Kadian-Dodov et al. [18]	Warchol-Celinska et al. 2020 [22]
Total Patients (N)	498 (Meta-Analysis), 117 (Institutional Series)	921	232
Gender of Patients	85% Female (Meta-Analysis)	93.5% Female, 6.5% Male	17.2% men, 82.8% women
Age	Range 4 to 83 years	Mean 52.2 ± 13.4 years	Mean 42.7 ± 15.7 years
Patients with Intracranial Aneurysms (N)	108 (Meta-Analysis); 28 (Institutional Series)	43 (21.5% of patients with aneurysms)	29 (12.5%)
Gender of Patients with Intracranial Aneurysms	25 Males, 103 Females (Meta-Analysis)	39 Females (90.7%), 4 Males (9.3%)	Not specified
Age of Patients with Intracranial Aneurysms	Mean age 60 years (Institutional Series)	Not specified	Not specified
Location of Aneurysms	- Anterior communicating artery: 9 - Posterior communicating artery: 7 - Ophthalmic artery: 4 - Internal carotid artery: 5 - Middle cerebral artery: 3 - Basilar tip: 2	- Anterior communicating artery: 12 - Posterior communicating artery: 7 - Basilar artery: 6 - Middle cerebral artery: 5 - Other locations (e.g., ICA, other branches): 13	specific locations not detailed
Rate of Multiple Aneurysms	21% (in patients with FMD and aneurysms)	Not specified in the study	Not specified
Size of Aneurysms	Not specified in the study	Not specified in the study	Not specified
Rate of SAH at Presentation	22 patients presented with symptoms, including 19 with SAH (Institutional Series)	8.4% of patients with aneurysms had a history of SAH	29/730 (4.0) in multifocal FMD group and 1/281 (0.4) in focal FMD group

## Data Availability

No new data were created in this paper.

## Abbreviations

CRF	Case Report Form
CTA	Computed Tomography Angiography
DSA	Digital Subtraction Angiography
FMD	FibroMuscular Dysplasia
IA	Intracranial Aneurysm
ICA	Internal Carotid Artery
MIP	Maximum Intensity Projections
MPR	Multiplanar Reconstruction
MRA	Magnetic Resonance Angiography
SAH	Subarachnoid Hemorrhage
UIA	Unruptured Intracranial Aneurysm
VA	Vertebral Artery
